# Controlling Antigen Fate in Therapeutic Cancer Vaccines by Targeting Dendritic Cell Receptors

**DOI:** 10.1021/acs.molpharmaceut.3c00330

**Published:** 2023-09-18

**Authors:** Zacharias Wijfjes, Floris J. van Dalen, Camille M. Le Gall, Martijn Verdoes

**Affiliations:** † Chemical Immunology group, Department of Medical BioSciences, Radboud University Medical Center, Geert Grooteplein Zuid 28, 6525 GA Nijmegen, The Netherlands; ‡ Institute for Chemical Immunology, Geert Grooteplein Zuid 28, 6525 GA Nijmegen, The Netherlands

**Keywords:** cancer vaccine, immunotherapy, cross-presentation, dendritic cells, endocytic receptor

## Abstract

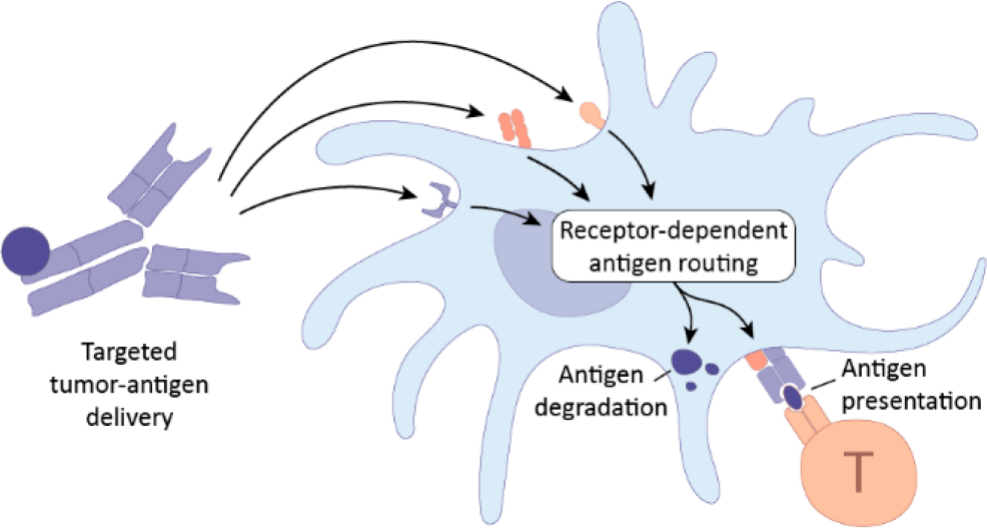

Antigen-presenting cells (APCs) orchestrate immune responses and are therefore of interest for the targeted delivery of therapeutic vaccines. Dendritic cells (DCs) are professional APCs that excel in presentation of exogenous antigens toward CD4^+^ T helper cells, as well as cytotoxic CD8^+^ T cells. DCs are highly heterogeneous and can be divided into subpopulations that differ in abundance, function, and phenotype, such as differential expression of endocytic receptor molecules. It is firmly established that targeting antigens to DC receptors enhances the efficacy of therapeutic vaccines. While most studies emphasize the importance of targeting a specific DC subset, we argue that the differential intracellular routing downstream of the targeted receptors within the DC subset should also be considered. Here, we review the mouse and human receptors studied as target for therapeutic vaccines, focusing on antibody and ligand conjugates and how their targeting affects antigen presentation. We aim to delineate how targeting distinct receptors affects antigen presentation and vaccine efficacy, which will guide target selection for future therapeutic vaccine development.

## 1 Introduction to Vaccines

Vaccines are immunological tools to boost the immune system of the recipient. The aim of prophylactic vaccination is to generate immunological memory after exposure to an antigenic challenge. Vaccines drastically improved global healthcare playing a crucial role in the eradication of smallpox and rinderpest, and controlling many other pathogenic diseases.^1,2^ In principle, a vaccine contains two fundamental components: antigens to confer specificity and adjuvants to induce antigen-presenting cell (APC) maturation and immunity.

APCs are key regulators of adaptive immunity. APCs take up and process antigens into epitopes that are presented via major histocompatibility complexes (MHCs). They express pathogen recognition receptors (PRRs) to discriminate between harmless and hazardous antigens.^3^ Engagement of PRRs such as toll-like receptors (TLRs), by damage associated molecular patterns (DAMPs) or pathogen associated molecular patterns (PAMPs) leads to APC maturation. Mature APCs downregulate antigen uptake, while enhancing antigen preservation and presentation on MHC.^4^ Moreover, APC maturation causes an increase in the expression levels of costimulatory molecules such as CD40, CD80 (B7–1), or CD86 (B7–2) and an inflammatory cytokine profile characterized by IL-2, IL-12, and IFN-γ.^5−7^ In this state, APCs license B cells, CD4^+^ T cells, and CD8^+^ T cells to induce an immune response against the presented antigen. Antigen uptake without engagement of PRRs causes the APC to present the antigen in an immature state, which can induce anergy in reactive CD4^+^ or CD8^+^ T cells or activate peripherally induced regulatory T cells (iTregs).^8^ Among APCs, dendritic cells (DCs) are considered the most specialized in antigen processing and presentation.

Researchers are investigating therapeutic vaccination strategies with the goal to replicate the success of prophylactic vaccines.^9−11^ Therapeutic cancer vaccines aim to induce or reactivate adaptive immune responses toward an established tumor. They are highly promising in the field of cancer immunotherapy, because they can induce antigen-specific memory responses that could prevent cancer relapse by maintaining long-term immune surveillance against cancer cells. The initial response of cancer vaccines is directed against a restricted set of antigens present in the vaccine. This can be either tumor-associated antigens (TAAs) or tumor-specific antigens (TSAs). TAAs are commonly overexpressed or aberrantly expressed proteins on tumors, for instance, glycoprotein 100 (gp100) or tyrosine related protein-2 (TRP-2). As TAAs are often shared across patients and cancer types, their incorporation in vaccines could benefit a larger group of patients and ease the manufacturing process. However, their efficacy is threatened by pre-existing central and peripheral tolerance, which could limit their immunogenicity. In contrast, TSAs originating from, for example, point or frame shift mutations are highly immunogenic. Since TSAs are unique to each patient, a personalized approach is necessary for their identification and production of the vaccine. This presents a manufacturing challenge. Second, tumors develop an immunosuppressive microenvironment, which could induce peripheral tolerance toward TSAs in the absence of strong immunostimulatory agents to drive the immune response. After the initial tumor cell killing by effector immune cells, other tumor antigens are released to induce epitope spreading. This can expand the range of tumor antigens recognized by the immune system and improves responses against the tumor.^12^

Two main strategies exist in therapeutic vaccination focused on antigen presentation by DCs: 1) Ex vivo DC therapy consists of activating and loading patient-derived DCs ex vivo and the subsequent transfusion of the matured DCs into the patient. 2) In vivo cancer vaccination depends on targeted delivery of antigens and immunostimulatory adjuvants to DCs in vivo. Subsequently, the trained DCs will activate antigen-specific responses via CD8^+^ cytotoxic and CD4^+^ helper T cells to elicit antitumoral immunity.^13^ The following paragraphs introduce these vaccination strategies and their associated advantages.

Ex vivo DC therapy relies on differentiating and maturing patient-derived DCs or DC progenitors ex vivo while pulsing them with tumor lysate or antigens. Tumor-primed DCs are transfused back in the patient and can migrate to the lymph nodes where they prime tumor-specific T cells.^14^ Most studies have relied on monocyte-derived DCs (moDCs), which are easily generated in large numbers. After over two decades of evaluation, it is evident that patients almost always respond to the cell therapy by generating vaccine-specific T cells but only show modest clinical benefits.^15−17^ A recent murine study demonstrated that host DCs are essential for T cell priming after vaccination with ex vivo loaded moDCs. It is theorized that most transfused moDCs cannot migrate to lymph nodes and die upon injection. Upon cell death, they release their tumor antigens to host DCs instead of priming T cells themselves. This suggests that moDCs are not the optimal choice for ex vivo DC therapies.^18,19^ Clinical studies have recently shown that the use of naturally present DCs outperforms moDCs.^20^ Additionally, it was demonstrated that ex vivo loaded type 1 conventional DCs (DC1s), but not type 2 conventional DCs (cDC2s) or moDCs, can drive tumor rejection in mice independently of host DCs.^21^ As such, DC1-based ex vivo DC therapy has an increased potential to function as cancer therapy.^22,23^ While DC1s are scarce in peripheral blood (<0.05% of PBMCs), technical advances such as cell reprogramming or differentiation from stem cells to generate larger numbers of functional DC1s will allow clinical studies utilizing autologous DC1s.^24−26^

**Figure 1:**
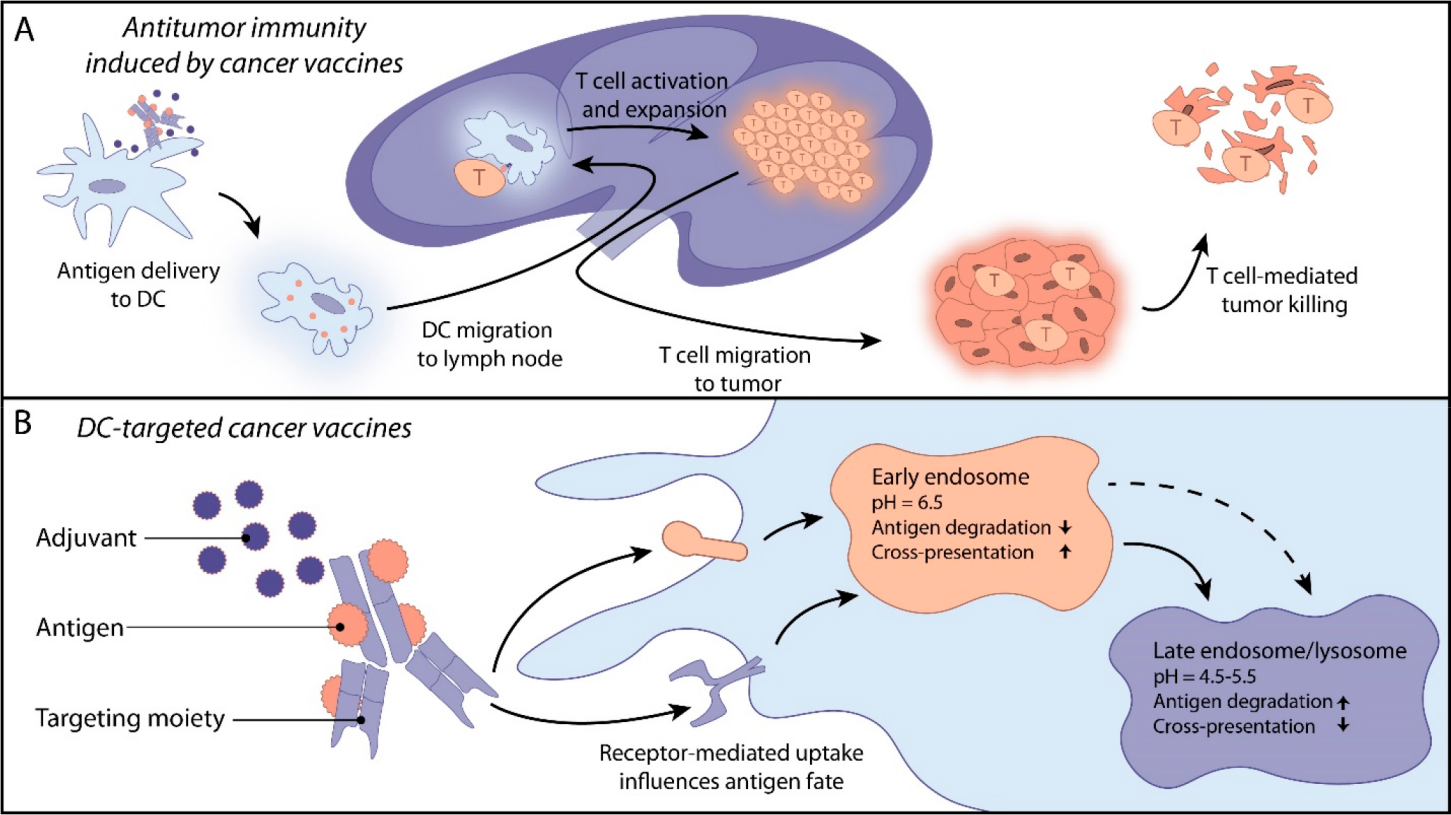
A) Schematic representation of mechanism of action from targeted antigen delivery to T cell mediated tumor killing. B) Schematic representation of DC-targeted cancer vaccines and the influence of receptor targeting on antigen fate. This review covers antibody-, antibody fragments, and nanobody and ligand conjugates. For image clarity only an antibody is depicted as a targeting moiety.

In vivo cancer vaccines deliver antigenic epitopes to APCs to elicit antitumor immunity (Figure 1a). This can be promoted by targeting APC surface molecules. TAAs or TSAs can be delivered as full proteins, peptides, or antigen-encoding nucleotides.^13^ Immunostimulatory adjuvants can be administered systemically or incorporated into the vaccine.^27−29^ Moreover, the antigen itself can be intrinsically immunostimulatory, such as antigen-encoding DNA or mRNA, that can bind to TLRs 3, 7, 8 or 9.^30^ The manufacturing of in vivo vaccines is relatively affordable and scalable and does not require the isolation and culturing of DCs from the patient. This could result in readily available off-the-shelf therapies and is a major advantage in comparison to ex vivo DC therapy.^31^ Several in vivo cancer vaccines are currently in clinical trials. NEO-PV-1 is a neoantigen peptide-based vaccine adjuvanted with poly-ICLC, which was demonstrated to be safe in a phase I study.^32^ Combination of NEO-PV-1 with nivolumab, an anti-PD-L1 immune checkpoint inhibitor, increased T cell reactivity and epitope spreading in a phase Ib study.^33^ Lipo-MERIT (BNT111, FixVac) and mRNA-4157 are mRNA-formulated tumor antigens encapsulated in liposomes. These mRNA/liposomes assemblies have demonstrated an acceptable safety profile, with promising immunological and clinical responses in phase I/II trials.^34−36^ Human papillomavirus-16 (HPV-16)-derived synthetic long peptides conjugated to Amplivant, a synthetic TLR2 agonist, were administered intradermally in a phase I study. This vaccine was capable of inducing T cell responses as measured by IFN-γ ELISpot assays.^29^ In this trial, most patients developed CD4^+^ T cell responses, while CD8^+^ T cell responses were observed less frequently. These recent trials showed promising results, yet none of these vaccines actively target APCs. This while the nonspecific uptake of these vaccines by other cells could decrease their effectiveness and could potentially cause harmful off-target responses.

DCs are ideal candidates for targeted vaccine delivery. Pioneering studies by Steinman and colleagues showed that selective delivery of antigens to DCs improves T cell priming.^37,38^ These results have been confirmed by a myriad of follow-up studies, which reinforces that targeting antigens toward DCs improves antigen presentation, adaptive immune responses and tumor rejection.^38,39^ DCs can engage CD4^+^ T helper cells as well as cytotoxic CD8^+^ T cells, both of which are required to induce long lasting antitumor immune responses.^40,41^

DCs display a large array of specialized surface receptors functioning as pathogen sensors. Downstream signaling of these receptors culminates in an immune response mechanistically biased to the elimination of the given pathogen. Sensing of viruses results in type I interferon release, which promotes cross-presentation and induces a CD8^+^ T cell-bias.^42^ Detection of bacteria or helminths induces a Th1- or Th2-bias, respectively. It may be possible to exploit these biases by selecting specific DC surface receptors to control the immune responses generated by vaccination. In line with this, preclinical studies have reported different immunological and clinical outcomes when comparing DC surface markers as vaccine target. This holds true for receptors expressed by distinct DC subpopulations and also for receptors expressed by the same DC subpopulation.^43−46^

Selecting specific cell surface targets can enhance the effectiveness of therapeutic vaccines by determining the subpopulation of DCs targeted, affecting the endosomal routing and antigen fate, and ultimately steering the immune response (Figure 1b).^47,48^ Decoupling the influence of the DC subset from that of the cell surface receptor is difficult, and few comparative studies have set out to investigate this issue. In this Review, we aim to delineate the impact of target receptor choice on the efficacy of DC-targeted therapeutic vaccines, in a perspective focused on endosomal routing inherent to the receptor. We set out to compare the effects of antibody and ligand conjugates on antigen presentation and immune response, particularly in the context of cancer vaccines. Ultimately, we aim to guide the target selection for the development of therapeutic cancer vaccines.

## 2 General Considerations on Immune Cells Involved in Therapeutic Vaccines and Antigen Processing Pathways

### 2.1 Types of Immune Cells Induced by Therapeutic Vaccines

#### 2.1.1 Dendritic Cells

DCs can be divided into several subpopulations: plasmacytoid DCs (pDCs) are involved in antiviral immunity via secretion of type I and type III interferons;^49^ DC1s are known to preferentially induce CD8^+^ T cell mediated immune responses but also can induce CD4^+^ T cells, such as T helper type 1 (Th1);^50,51^ DC2s represent 90% of the conventional DCs (cDCs) and excel in the induction of CD4^+^ T cells;^52,53^ DC3s, macrophages, and Langerhans cells can also scavenge antigens.^54^ DC3s have only recently been described as separate subset and are mainly considered to be immunosuppressive.^55^ In such, they might hamper the efficacy of vaccines that target DC subsets with an overlapping receptor expression.^56^ As discussed above, moDCs are DC-like cells often used as in vitro models in DC biology, but they are also present in circulation during the course of inflammation. The unique expression profile of receptors on DC subpopulations enables targeting of specific subtypes with specialized properties and determines the encountered endocytic pathway (Table 1). This offers an opportunity to steer the immune system toward the desired response.

**Table 1:**
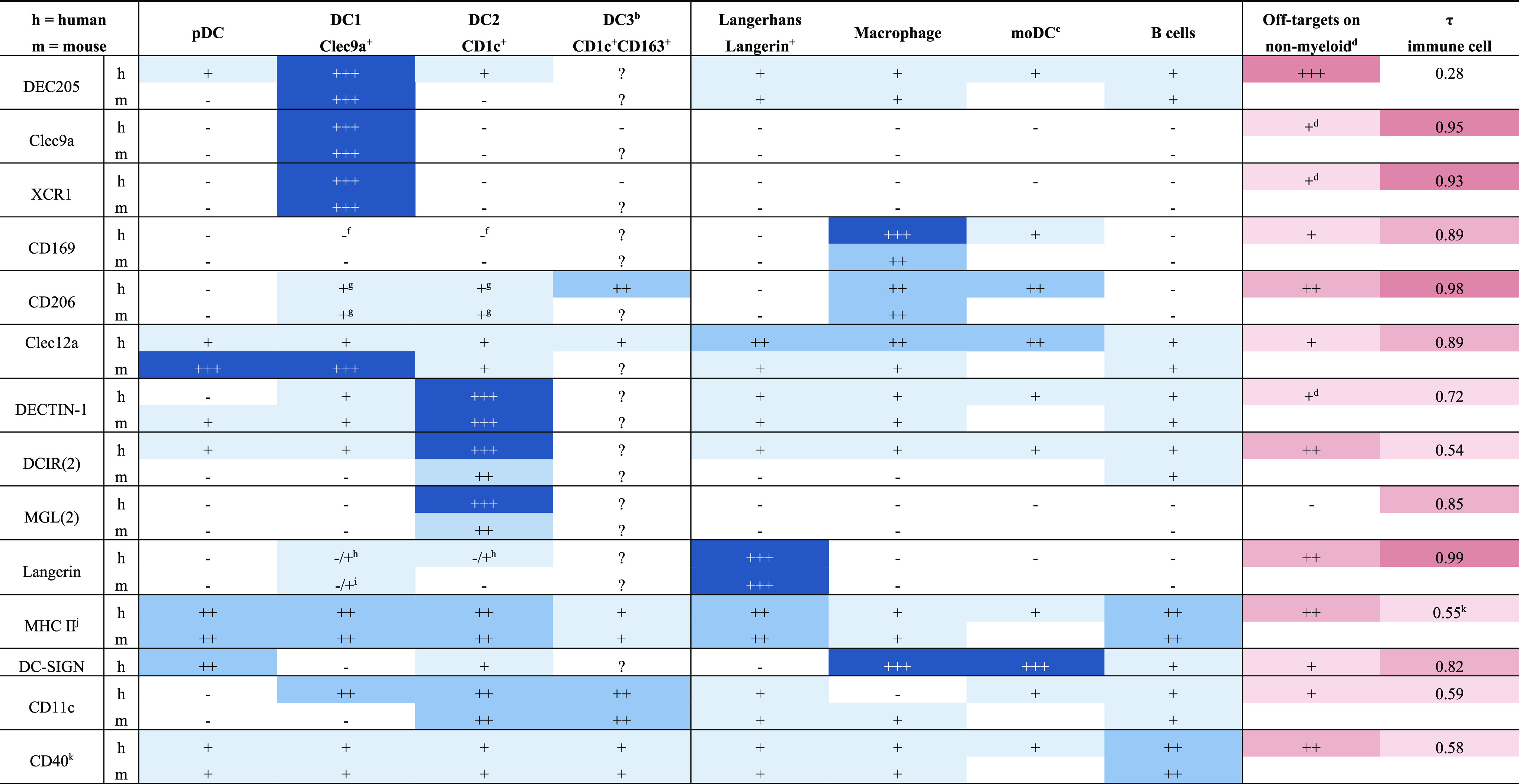
Receptor Expression on Human and Mouse APCs of the Main Vaccine Targets Discussed in This Review^a^

a The relative expression is indicated by −, +, ++, and +++.

b Receptor expression on DC3s is not yet studied for cancer vaccine targets but expected to be comparable to DC2s.

c Murine equivalent of moDCs are poorly defined; thus, no receptor expression could be determined.

d Receptor expression is determined by the protein atlas.^57^ Tissue specificity index (τ) of receptor expression in human immune cells as determined by the protein atlas. τ ranges between 0 and 1, where 0 indicates broad expression of the receptor on different immune cells and 1 indicates highly specific immune cell type expression.^58^ No such database exists for receptor expression in mouse.

e Main off-target on nonmyeloid cells are Schwann cells.

f CD169 is expressed on the pre-DC progenitor of cDCs.

g CD206 is downregulated upon APC maturation.

h Conflicting results have been published.

i Langerin expression is mouse strain-dependent.

j MHC II is upregulated upon DC maturation.

k Multiple genes exist for the different MHC types in human: CIITA is used as representative gene for MHC II expression, because expression of all MHC types is under control by CIITA.

l Expression of CD40 is upregulated on mature APCs.

#### 2.1.2 CD8^+^ T Cells

MHC I restricted T cell responses have received the most attention in cancer vaccine design. CD8^+^ T cells can lyse tumor cells directly by releasing cytotoxic granules or by inducing apoptosis via the Fas/FasL pathway.^59^ Incorporation of CD8^+^ T cell-specific epitopes in cancer vaccines is feasible. Epitopes are well described for the most common TAAs in multiple MHC subclasses, and algorithms have been developed to predict putative MHC I epitopes originating from TSAs. Either a too strong or too weak antigenic stimulation during the initial priming can drive CD8^+^ T cell dysfunction and lack of tumor control.^60^ The potential impact of the strength of TCR stimuli on CD8^+^ T cell responses is currently not understood fully.

#### 2.1.3 CD4^+^ T Cells

CD4^+^ T helper cells provide support to other immune cells during priming and activation by producing large amounts of immunostimulatory cytokines. In the context of antitumor responses, CD4^+^ T cells license DCs and improve priming of CD8^+^ T cells,^40^ whereas CD8^+^ T cell priming in absence of CD4^+^ T cells leads to T cell exhaustion.^61^ Moreover, CD4^+^ T cells support B cell development and affinity maturation. CD4^+^ T cells have repeatedly been shown to be crucial for antitumor responses.^62,40^ Results of a trial using peptides selected to encompass MHC I TSAs adjuvanted with poly-ICLC noted that most patients mounted TSA-specific CD4^+^ T cells responses instead of the expected CD8^+^ T cell responses.^63,64^ We suggest that the predisposition of CD4^+^ T cells to respond to tumors should be harnessed by including MHC II epitopes in vaccination strategies.^40^ However, MHC II epitopes are often insufficiently characterized for TAAs. Algorithms to identify TSA-derived MHC II epitopes are not as accurate due to less stringent factors determining the binding of MHC II epitopes in comparison with MHC I epitopes. To bypass the need to identify MHC II epitopes, Sahin and colleagues have postulated that point mutations are intrinsically immunogenic and aimed to generate a diverse pool of epitope variants by placing mutations strategically within epitopes.^65,66^ Yet, the elimination of MHC II-deficient tumors is still dependent on CD4^+^ T cells.^62^ These findings indicate that CD4^+^ T cells may not necessarily need to recognize tumor-specific antigens.^67^ Instead, pre-existing memory CD4^+^ T cells directed against universal epitopes, such as those found in diphtheria and tetanus, might provide the necessary support to the immune system. Therefore, the inclusion of universal epitopes in cancer vaccines could enhance antitumoral effectiveness while simplifying the manufacturing process.

#### 2.1.4 B Cells

It remains elusive whether humoral immunity to cancer antigens is important for antitumor responses. In principle, antibodies can recognize antigens aberrantly expressed by tumor cells and mediate antibody-dependent cytotoxicity by natural killer (NK) cells or phagocytosis by macrophages. Tumor-reactive antibodies also promote antigen uptake by DCs via immune complexes and broaden the tumor-reactive T cell repertoire.^68,69^ Nevertheless, humoral immunity is often overlooked in cancer vaccination strategies as well as the antigen-presenting capacity of B cells.

### 2.2 Antigen Cross-Presentation

**Figure 2:**
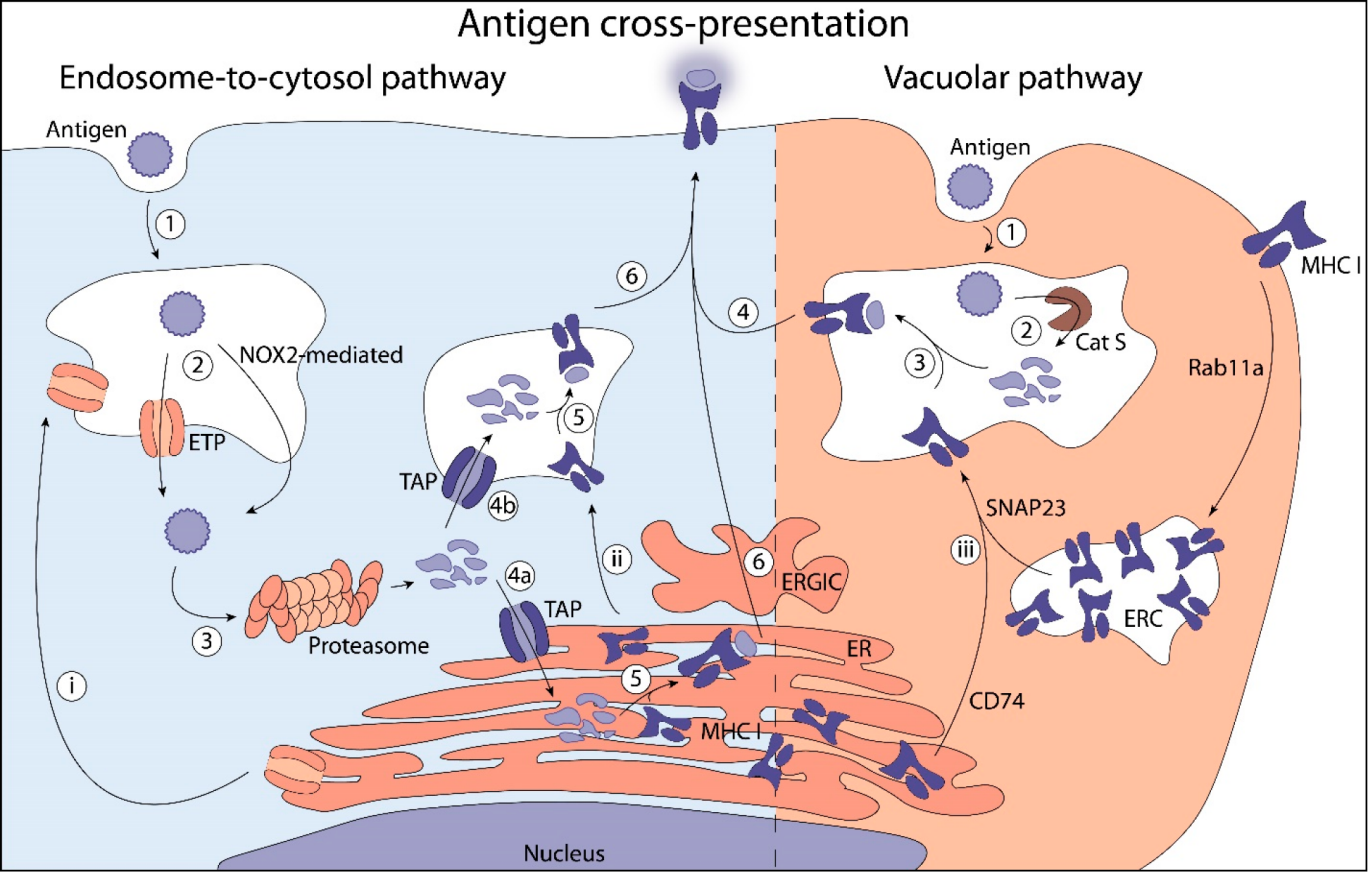
Overview of the XP pathways. Endosome-to-cytosol pathway: 1) Antigen uptake by endocytosis or phagocytosis. 2) Endosomal escape is mediated by active transport through ETPs as HRD1, Derlin-1, and Sec66b or through lipid peroxidation mediated by NOX2.^75−78^ 3) Cytosolic cleavage of antigen by proteasome. 4) TAP-mediated uptake of antigen into the ER (a) or endosomes (b). 5) Loading of antigen onto MHC I. 6) Transport of MHC I to the cell surface for antigen presentation. (i) Transport of ETPs originating in the ER to endosomes is most likely mediated by Sec22b.^79^ (ii) MHC I originating in ER are transported to endosomes. Vacuolar pathway: 1) Antigen uptake by endocytosis or phagocytosis. 2) Proteolytic processing of antigen in early endosomes is presumably mediated by cathepsin S.^83^ 3) Loading of antigen onto MHC I. 4) Transport of MHC class I to the cell surface for antigen presentation. (ii) MHC I is transported to endosomal recycling compartments (ERC) from cell surface mediated by Rab11a and subsequently SNAP23 to fuse with endosomes, or MHC I is transported to endosomes from the ER mediated by CD74.^84,85^

Antigenic epitopes are present to T cells on DC surfaces via MHC molecules. In principle, exogenous antigens are presented via MHC II to CD4^+^ T cells, whereas endogenous antigens are presented via MHC I to CD8^+^ T cells.^70^ Yet, exogenous antigens can be presented on MHC I, a process referred to as cross-presentation (XP).^71−73^ XP is crucial to inducing a cytotoxic immune response against virally infected or malignant cells. In particular DC1s excel at XP and subsequent induction of cytotoxic immune responses.^74^ The current paradigm proposes two main pathways for XP (Figure 2).

#### 2.2.1 Endosome-to-Cytosol Cross-Presentation

The endosome-to-cytosol pathway describes the escape of antigens from endocytic compartments to the cytosol, their processing by the proteasome, and the transport to the endoplasmic reticulum (ER) or endosomes, where epitopes are loaded onto MHC I. Antigen escape can be passive, following membrane lipid peroxidation induced by NOX2;^75^ or active, via endosome transporter proteins (ETPs) such as Sec61, Derlin-1, and HRD1.^76−78^ Sec22b likely regulates antigen transport by ETPs.^79^ After they escape the endocytic compartment, antigens are processed by the ubiquitin-proteasome system (UPS), an elaborate catalytic machinery able to efficiently hydrolyze cytosolic and nuclear proteins. Antigenic peptides released by the proteasome are translocated to the ER or endosomes by the transporter associated with antigen processing (TAP). The endosome-to-cytosol pathway strictly requires TAP activity.^80,81^ In the ER or endosomes, antigenic epitopes are loaded onto MHC I and transported via the Golgi apparatus to the cell surface.

#### 2.2.2 Vacuolar Cross-Presentation

The vacuolar pathway is TAP- and proteasome-independent.^82^ In this second main XP pathway, antigen processing and epitope loading take place directly inside early endosomes. Cathepsin S is presumably largely responsible for the antigen processing, as its inhibition leads to major deficiencies in the vacuolar pathway.^83^ MHC I is transported to endosomes in vesicles originating from the ER under regulation of CD74, or via endosomal recycling compartments (ERCs) under control of Rab11a and SNAP23.^84,85^ The recruitment of MHC I from the ERCs is TLR-dependent, and promoted by the simultaneous presence of TLR stimulating signals and antigens inside individual phagosomes.^86−88^ This is thought to be a mechanism to transiently enhance XP in response to an antigenic threat.

## 3 Promoting Antigen Presentation on MHC I to Induce Cytotoxic Immunity

In the next sections, we introduce receptors described to target therapeutic vaccines to DCs. For each receptor, we highlight what has been reported in murine and human studies and briefly summarize the influence of targeting this receptor on the downstream response.

### 3.1 DEC205

DEC205 (CD205, LY75, Clec13b) binds keratin at slightly acidic pH to recognize apoptotic or necrotic cell debris.^89−91^ DEC205 can mediate the internalization and trafficking to late endosomes/lysosomes of CpG oligonucleotides promoting TLR9 signaling.^92^ In the mouse, DEC205 is expressed mainly by CD8^+^ DCs (DC1s). In humans, besides its expression on DC1s, DEC205 is also expressed at low levels on DC2s and pDCs, and in high levels on epithelial cells (Table 1).^93,94^ In a pioneering work, Steinman and colleagues targeted antigens to DEC205 and established that delivering antigens specifically to DCs could enhance antigen-specific T cell responses and improve the therapeutic window.^38,39^

#### 3.1.1 DEC205–Mouse

Targeting of mouse DEC205 is extensively described in literature. In Steinman’s initial studies, ovalbumin (OVA) protein was chemically conjugated to the anti-DEC205 monoclonal antibody (mAb). Conjugation of OVA to anti-DEC205 increased CD8^+^ T cell responses over 1000-fold, and CD4^+^ T cell responses are over 50-fold compared to soluble OVA.^39^ Co-administration of immunostimulatory anti-CD40 mAb was shown to be crucial for tumor rejection and prevented the induction of tolerance.^38^ These promising results encouraged further study of the DEC205 receptor: Many studies have reported enhanced XP of TAAs (gp100, HER2/neu, mesothelin) and strong CD8^+^ T cell responses upon targeting DEC205.^95−97^ Antigens targeted to DEC205 mainly accumulated in late endosomes, which should bias antigen processing toward proteolysis, MHC II presentation, and CD4^+^ T cell activation.^98^ MHC II antigen presentation was not increased as much as XP. We attribute the enhanced CD8^+^ T cell activation to the combination of DEC205-mediated intracellular trafficking with an increased antigen uptake by DC1s, which upregulate Tap1/2 and Sec61 thereby promoting the endosome-to-cytosol XP pathway.^45,81^

#### 3.1.2 DEC205–Human

The broad expression profile of DEC205 in human cells complicates interspecies comparison.^93^ The first in-human targeted cancer vaccine consisted of an anti-DEC205 mAb fused to the cancer testis antigen NY-ESO-1 adjuvanted with coadministered poly-I:C (CDX-1401). Its clinical evaluation in phase I and II trials demonstrated a favorable safety profile and moderate CD4^+^ and CD8^+^ T cell responses without induction of Tregs.^99,100^ Pretreatment with systemic fms-like tyrosine kinase 3 ligand (Flt3l) 1 week before CDX-1401 administration increased the abundance of DCs prior to vaccination and improved T cell responses to CDX-1401.^101^ This preliminary study did not examine the specific contribution of different DC subsets to T cell activation. In a different study, PBMCs isolated from NY-ESO-1-expressing cancer patients were treated with anti-DEC205 or anti-CD206 mAbs fused to NY-ESO-1.^102^ While receptor targeting improved expansion of antigen specific CD4^+^ and CD8^+^ T cells in vitro, no significant differences in targeting DEC205 or CD206 were observed. Targeting influenza peptide to isolated human DC1s in vitro via DEC205 resulted in lower levels of XP in comparison with targeting to CD40 and CD11c, both known to direct antigens to early endosomes. Strikingly, early endosomal targeting resulted in comparable levels of XP in DC1s and DC2s. This emphasizes the importance of taking into account antigen routing to optimize processing in cancer vaccines.^47^ In total, ten clinical trials using CDX-1401 have been initiated (Table 2). These pioneering trials in DEC205-targeted antigen delivery could provide valuable insight into the translation of targeted vaccines. Yet, targeting human DEC205 might not be as effective at inducing antigen XP and subsequent CD8^+^ T cell activation due to routing to late endosomes. Moreover, the broad expression profile of DEC205 in myeloid and nonmyeloid human cells may result in undesirable off-targeting and could decrease the efficacy of DEC205 targeted vaccines in vivo.

**Table 2:**
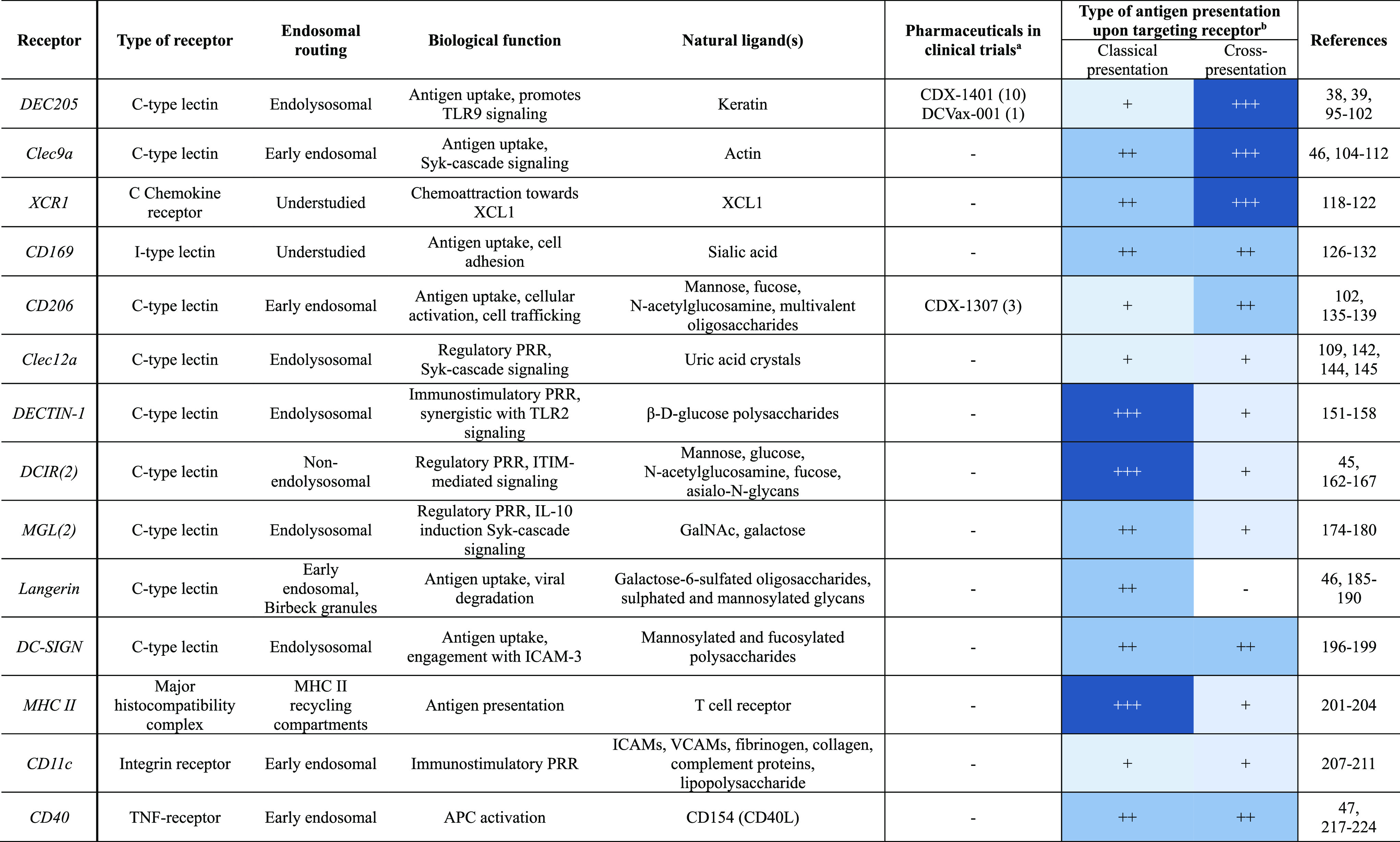
Comprehensive Overview of the Receptors Discussed in This Review^38,39,45−47,95−102,104−112,118−122,126−132,135−139,142,144,145,151−158,162−167,174−180,185−190,196−199,201−204,207−211,217−224^

a Clinical trial identifiers: *CDX1401*: NCT03358719, NCT01834248, NCT01522820, NCT02661100, NCT02129075, NCT03206047, NCT02413827, NCT02495636, NCT02166905, NCT00948961; *DCVax-001*: NCT01127464; *CDX-1307*: NCT00648102, NCT01094496, NCT00709462.

b The relative propensity of antigen presentation is indicated by −, + , ++, or +++, based on the studies described in this review.

c Cross-presentation by targeting CD169 occurs indirectly through antigen transfer to DCs.

### 3.2 Clec9a

Clec9a (DNGR-1, CD370) recognizes actin exposed to damaged cells. It binds filamentous actin in complex with cytoskeletal proteins and signals through the Syk-cascade.^103^ Clec9a is selectively expressed on DC1s in mouse and human (Table 1).^104^ Clec9a and DEC205 differ in their intracellular routing. In an in vitro study, fluorescently labeled anti-DEC205 mAbs but not anti-Clec9a mAbs colocalized with lysotracker. Instead, anti-Clec9a colocalized with necrotic cell material away from lysosomes.^105^ In addition, engagement of Clec9a signals for phagosomal rupture, resulting in antigen escape into the cytosol, which, in turn, enhances XP. These features make Clec9a a compelling target to promote XP.

#### 3.2.1 Clec9a–Mouse

In B6 mice, delivery of CD8^+^ and CD4^+^ T cell epitopes to DC1s using anti-Clec9a mAb results in enhanced XP and MHC II presentation, respectively, in comparison with untargeted controls.^104,106^ In CB6.F1 mice, HIV gag-p24 peptide targeted to either Clec9a or DEC205 resulted in similar CD8^+^ T cell proliferation and comparable IFN-γ levels in an restimulation assay.^46^ Targeting antigens with CBP12, a 12-mer peptide specific to Clec9a, also demonstrated enhanced XP and increased specific T cell responses.^107,108^ CBP12-mediated delivery of the peptides of the peptides of the OVA_257–264_ and gp100 under adjuvant-free conditions demonstrated potent antitumor responses in anti-PD1-resistant B16-OVA and B16 melanoma, respectively. Mechanistically, the authors demonstrated that CBP12 binding triggered IL-21 signaling, which was crucial for vaccine activity. Targeting Clec9a with mAb-OVA fusion constructs increased XP in comparison with Clec12a targeting. Targeting Clec9a significantly enhanced humoral responses compared to DEC205 and Clec12a, even in adjuvant-free conditions.^109^ Altogether, this indicates that Clec9a is a promising target for the delivery of antigen to murine DC1s.

#### 3.2.2 Clec9a–Human

CLEC9a has restricted expression on human DC1s, unlike DEC205. Viral antigen (pp65), NY-ESO-1 polypeptide, or Wilms’ tumor 1 antigen delivery to Clec9a or DEC205 was compared using human PBMCs.^110−112^ Naïve and memory CD8^+^ T cell activation was enhanced upon antigen delivery to Clec9a compared with DEC205. Yet, in humanized NSG-A2 mice, equally potent XP of pp65 was observed for both targets.^110^ Unlike humans, NSG mice express do not express DEC205 on nonlymphatic endothelial cells. The authors speculate that this leads to a higher persistence of anti-hDEC205 mAb in NSG mice than is expected in humans, effectively increasing the specificity of anti-hDEC205 to DC1s. Due to the highly specific expression profile of Clec9a on human DC1s, it is expected that a Clec9a-targeted vaccine will demonstrate an increased activity compared to DEC205 in a clinical setting.^113^

### 3.3 XCR1

X-C motif chemokine receptor 1 (XCR1, GPR5) mediates chemoattraction toward lymphotactin (XCL1), a chemokine secreted by activated T cells and NK cells.^114,115^ This interaction promotes antigen uptake in inflammation sites and interaction with CD8^+^ T cells.^116^ Similar to Clec9a, XCR1 expression is restricted to DC1s (Table 1).^114,116,117^ Because of the lack of publicly available mAbs against human XCR1, studies on XCR1 have been limited to XCL1-mediated targeting.

#### 3.3.1 XCR1–Mouse

XCL1 fused to OVA or influenza antigens induces strong CD8^+^ T cell cytotoxicity in B6 mice.^118,119^ Fossum and colleagues performed a thorough comparative study of XCR1, Clec9a and DEC205 as potential DC1 vaccine targets.^118^ Immunization with plasmid DNA encoding XCR1 or Clec9a targeted hemagglutinin (HA) protected mice in a lethal viral challenge, whereas DEC205 targeted HA did not. Clec9a and DEC205 were targeted by nanobodies, whereas XCR1 was targeted via XCL1. The strongest immune responses were noted for XCR1, irrespective of the antigen or the mouse strain. This study is one of the few that compared the activities of vaccines targeting receptors specifically expressed by the same DC subpopulation. Future studies should confirm whether this also holds true for protein-based immunization and investigate the influence of the adjuvant. Taken together, these results emphasize that intrinsic factors of receptors, such as expression levels and endosomal routing, influence antigen presentation and subsequent T cell activation.

#### 3.3.2 XCR1–Human

XCL1–antigen fusions induced stronger antigen XP in comparison with free antigen or vehicle control in human PBMCs.^120−122^ Human XCL1–antigen fusions conserved its targeting ability in vivo, as confirmed in transgenic mice expressing human XCR1, and induces potent CD8^+^ T cell activation.^120^ Moreover, XCL1-NY-ESO-1-peptide-PEG_5k_ constructs retained their activity as DC1 chemoattractant, which could potentiate DC1 activation and XP by attracting DC1s toward the injection site.^121^ In summary, XCL1 has chemotactic properties and retains its DC1-specific targeting capability upon C-terminal modification. Further engineering of XCL1 constructs to increase its affinity, stability and agonistic activity could yield optimized targeting agents to explore in a therapeutic setting.^119,123^

### 3.4 CD169

The sialic acid binding immunoglobulin-type lectin 1 (SIGLEC-1, Sn, CD169) is involved in uptake and presentation of dead cell-associated antigens, including tumor antigens.^124^ CD169 is expressed on a subpopulation of macrophages located in the marginal zone of the spleen and the lymphatic sinuses of secondary lymph nodes. This strategic location allows CD169^+^ macrophages to filter and capture circulating antigens, pathogens or cellular debris.^125^ CD169^+^ macrophages act as an antigen reservoir for the splenic and sinusoidal resident lymphohematopoietic systems by retaining and releasing these antigens gradually. CD169 itself mediates antigen transfer to DCs, and especially DC1s, by binding to sialic acid containing glycans on the surface of DCs.^126^

#### 3.4.1 CD169–Mouse

Targeted delivery using anti-CD169 or CD169-specific ligands conjugated to TAA peptides resulted in antitumor responses in B6 mice.^127,128^ Mice that lack DCs but not CD169^+^ macrophages were as capable as WT mice at priming CD8^+^ T cells upon CD169-mediated delivery of OVA peptide.^129^ In a more physiological setup, CD169^+^ macrophages transferred antigens to DC1s to promote cross-priming of CD8^+^ T cells in vivo.^126^ This plausible mechanism may be exploited to improve the XP in targeted therapeutic vaccines.

#### 3.4.2 CD169–Human

Antigen delivery to CD169 resulted in a slower uptake compared to antigen delivery to DC-SIGN in moDCs.^128^ This correlated with murine data and hints toward a pathway favoring antigen retention for transfer to DC1s.^130^ Delivery of the tumor antigens gp100 and WT1 using anti-CD169 resulted in specific CD8^+^ T cell expansion possibly mediated through cross-talk between CD169^+^ macrophages and DC1s.^131^ Sialic acid covered liposomes were shown to induce antigen-specific CD8^+^ T cell activation and proliferation in human PBMCs.^132^ Targeting CD169^+^ macrophages could be an efficient way of indirectly targeting the relatively low number of DC1s by harnessing the higher prevalence of CD169^+^ macrophages and their capacity to retain antigens.

### 3.5 CD206

CD206 (MR, MCR1, MMR) is involved in numerous processes such as cellular activation, clearance of glycosylated molecules, promotion of antigen presentation, cell trafficking and collagen internalization.^133^ CD206 binds to mannose, fucose, and *N*-acetylglucosamine, but it is especially known for its high affinity for multivalent oligosaccharides.^134^ In DCs, soluble antigen uptake by CD206 leads to transport to early endosomes, which increases XP via the vacuolar pathway.^135,136^

#### 3.5.1 CD206–Mouse

CD206 appears to be involved in the uptake of soluble antigens by DCs. Mannan, an inhibitor of CD206-mediated endocytosis, completely blocked uptake of soluble OVA in mouse bone marrow-derived DCs (BMDCs) but not of cell-associated OVA. In line with this, OVA-specific CD8^+^ OT-I cells activation upon vaccination with OVA was hampered in CD206^–/–^ B6 mice.^135^ This finding prompted a study on trafficking and processing of OVA in BMDCs of WT or CD206^–/–^ B6 mice.^136^ MHC II antigen presentation of the CD4-epitope was unaltered in CD206^–/–^ BMDCs, whereas XP of the CD8-epitope was strongly reduced in CD206^–/–^ BMDCs, as measured by OT-II and OT-I cell proliferation, respectively. Microscopy revealed that CD206-mediated uptake delivered antigens toward early endosomes and away from lysosomes, thus facilitating antigen preservation and XP. Mannosylation of antigenic peptides enhanced XP in B6 mice and resulted in stronger T cell proliferation compared to nonmannosylated peptides.^137^ This was not observed in CD206^–/–^ B6 mice, indicating a functional dependency on CD206.

#### 3.5.2 CD206–Human

Human chorionic gonadotropin β (hCG-β) is a TAA that is expressed in epithelial cancers. The targeted cancer vaccine CDX-1307, anti-CD206 mAb fused to hCG-β, demonstrated tolerable safety profiles in a phase I trial of bladder cancer patients. Patients developed T and B cell responses even if they presented hCG-β high serum levels pretreatment. This was especially pronounced upon coadministration of the TLR-agonists resiquimod and/or poly-ICLC.^138,139^ These trials demonstrate the feasibility of breaking tolerance against a self-antigen by targeting CD206. Unfortunately, the subsequent phase II trial (NCT01094496) was terminated due to portfolio prioritization. An in vitro comparative study of NY-ESO-1 delivery via anti-CD206 or anti-DEC205 was conducted using moDCs and patient-derived T cells.^102^ Targeting to either DEC-205 or CD206 increased XP of NY-ESO-1 compared to untargeted protein, but the MHC II presentation was comparable in all conditions. This suggests that targeted delivery of NY-ESO-1 to late (via DEC-205) or early (via CD206) endosomal compartments increased XP. These findings should be confirmed using isolated DC1s and DC2s in a similar setup. Of note, CD206 is highly expressed on tumor-associated macrophages (TAMs), which opens up the possibility to repolarize TAMs by targeting TLR7/8 agonists toward CD206.^140^ Simultaneous antigen delivery to TAMs is two-edged: If repolarization is successful, then it could remodel the TME and harness the antigen-presenting capacity of prevalent macrophages. However, if repolarization is unsuccessful, then it could strengthen peripheral tolerance.

### 3.6 Clec12a

Clec12a (MICL, CD371, DCAL-2) is an inhibitory PRR that recognizes uric acid crystals formed upon release of intracellular uric acid by dying cells.^141^ Clec12a inhibits the Syk-cascade and hampers ROS production to limit inflammatory responses. Clec12a is highly expressed on murine pDCs and DC1s, but equally expressed among human DC subpopulations (Table 1).^142,143^

#### 3.6.1 Clec12a–Mouse

Murine splenic DCs targeted ex vivo with anti-Clec12a mAb-OVA fusions displayed superior cross-presenting capabilities in comparison with Clec9a-targeting fusions as measured by proliferation of OT-I cells.^144,145^ Yet, in a head-to-head comparison of OVA protein fused to anti-Clec12a mAb, anti-Clec9a mAb, or anti-DEC205 mAb, Clec12a-targeting fusions induced lower proliferation of CD8^+^ T cells in B6 mice compared to the latter two.^109^ This emphasizes that in vitro studies of XP do not always accurately predict in vivo efficacy. CD4^+^ T cell proliferation was not observed for Clec12a-targeting fusions, whereas fusions targeting Clec9a or DEC205 resulted in both CD8^+^ and CD4^+^ T cell proliferation.^109^

#### 3.6.2 Clec12a–Human

Both Clec12a and DEC205 direct antigens to lysosomes via early endosomes. The retention of antigen in early endosomes has been shown slightly longer upon targeting Clec12a, thus promoting XP.^142^ Clec12a-mediated delivery of keyhole limpet hemocyanin (KLH) to moDCs, pDCs, DC2s, or DC1s led to enhanced XP, IFN-γ production, and CD4^+^ T cell proliferation compared to untargeted controls.^142^ These findings indicate a potential use for Clec12a as a target in cancer vaccines. However, mouse studies demonstrate no to little beneficial effects and challenge these conclusions.^109^ In summary, conflicting results prevent one from concluding on the potential of Clec12a as target to enhance XP.

## 4 Promoting MHC II Antigen Presentation to Improve CD4^+^ T Helper Responses

### 4.1 DECTIN-1

DECTIN-1 (Clec7a, CD369) initiates the release of antifungal cytokines and chemokines upon recognition of β-d-glucose polysaccharides, often present on pathogens.^146−148^ Its signaling is inherently immunostimulatory and is synergistic with TLR2 signaling.^149,150^ DECTIN-1 can be used to target DC2s (Table 1).

#### 4.1.2 DECTIN-1–Mouse

Notable differences in T cell activation were observed upon delivery of the OVA using anti-DECTIN 1 and anti-DEC205. Targeting DECTIN-1 increased CD4^+^ T cell proliferation and antibody production, whereas targeting DEC205 increased CD8^+^ T cell proliferation.^151^ β-glucan functionalization of nanoparticles increased CD4^+^ T cell proliferation compared to untargeted controls, and reduced tumor growth by elicitation of CD4^+^ Th1 cells, and to a lesser extent, of CD4^+^ Th9 cells.^152−155^ However, the dependency on DECTIN-1 remains to be demonstrated as particle functionalization can alter charge, uptake properties, and pharmacokinetics.

#### 4.1.3 DECTIN-1–Human

In humans, targeting DECTIN-1 via β-glucans or mAbs was shown to be immunostimulatory.^156,157^ This finding has been used to target moDCs with MART-1 peptide without extra adjuvant to activate T cells in vitro.^156^ The selective expansion of CD4^+^ Th17 cells in vitro may be explained by the involvement of DECTIN-1 in antifungal immunity.^158^ The intrinsic immunostimulatory capacity of β-glucans is being explored in phase I clinical trials to enhance the efficacy of anti-GD2 immunotherapy against neuroblastoma.^157^ The dual use of β-glucans as both DECTIN-1-targeting and immunostimulatory adjuvants should be further explored in cancer vaccine formulations.

### 4.2 DCIR(2)

DCIR (Clec4a, CD367) regulates inflammation and T cell immunity.^159^ The mouse homologue DCIR2 is uniquely expressed on DC2s, whereas DCIR is expressed less restrictively in humans (Table 1).^45^ DCIR(2) binds mannose, glucose, *N*-acetylglucosamine, fucose, and asialo-*N*-glycan(s). The latter is crucial in DC regulation processes.^160^ A single glycosylation site in its carbohydrate binding pocket controls the specificity of DCIR for its ligand.^161^

#### 4.2.1 DCIR2–Mouse

Dudziak and colleagues compared DCIR2 and DEC205 as vaccine targets.^45^ In vivo delivery of the OVA protein to DCIR2 resulted in a faster class switching to IgG2b/c, indicating a Th1-oriented response in B6 mice, whereas delivery to DEC205 increased antigen presentation on MHC I. Both vaccines elicited similar CD4^+^ T cell responses. Even though DEC205 targeting formulations led to increased CD8^+^ T cell responses, both formulations were capable of protection in a tumor challenge.^162^ In contrast, in BALB/c mice, immunization with viral NS1 protein delivered to DCIR2 did not provide protection in a lethal challenge, whereas delivery to DEC205 conferred partial protection.^163^ This reflects a heavy dependence on CD8^+^ T responses during a dengue challenge, while antitumor immunity benefits from the combined action of CD4^+^ and CD8^+^ T cells.^40,62^ It is possible that the strain in which these studies were made influenced the nature of the immune response, as B6 mice are Th1 prone while BALB/c are Th2-prone.^164,165^ Performing similar experiments in CB6.F1 mice (B6 × BALB/c (H2K^b+/d+^)) could help unify these findings. In addition, DCIR2 and DEC205 are expressed on distinct DC subsets, and it is thus not possible to dissect the effect of the receptor and routing from the inherent physiological differences among the DC subsets. Nevertheless, these studies demonstrate the possibility of directing the immune response by targeting different DC receptors.

#### 4.2.2 DCIR–Human

The less restricted expression of DCIR in humans enables a comparison of antigen delivery to the same receptor on different subsets. Myeloid DCs, pDCs, and Langerhans cells isolated from human PBMCs showed similar CD8^+^ T cell priming ability when targeted with anti-DCIR-MART-1 peptide or anti-DCIR-influenza matrix protein fusions.^166^ DCIR had a different intracellular routing than other C-type lectins and did not preferentially colocalize with either early, late, or recycling endosomes, nor with the ER or Golgi.^167^ In line with the poor association with the endolysosomal system, low CD4^+^ T cell responses were measured upon ligand internalization. These results should be translated with caution, as DCs might act differently in an in vivo setting.

### 4.3 MGL(2)

MGL (Clec10a, DC-ASGPR, CD301) is a galactose-binding lectin that is mainly targeted using its ligands, α- or β-linked *N*-acetyl galactosamine (GalNAc) or galactose.^168−170^ Activation of MGL leads to production of IL-10 via Syk signaling.^171^ MGL is primarily expressed on DC2s and downregulated upon DC activation.^172^

#### 4.3.1 MGL2–Mouse

MGL1 and MGL2 homologues have been identified in mice.^173^ Murine MGL2 can be specifically targeted by modifying antigens with GalNAc, which has been shown to enhance CD4^+^ T cell responses.^174,175^

#### 4.3.2 MGL–Human

Abnormal *O*-glycosylation of mucins is a trait often shared by tumors. The Thomsen-nouveau (Tn) antigen, α-linked GalNAc carried on serine or threonine residues, is often found on mucin-type glycoproteins such as MUC1 at the surface of carcinoma cells.^176^ Because MUC1 is a tumor antigen, the capacity of Tn to bind human MGL has been used to increase TAA delivery to DCs.^177−179^ Tn-MUC1 glycoprotein was detected only in MHC II compartments, while a short Tn-MUC1 glycopeptide was found in both MHC I and MHC II compartments. This suggests that antigen size or physicochemical properties can influence intracellular routing and that using short peptide antigens could favor XP. This remains to be confirmed with an increased sample size.^178^ As Tn-glycosylation can negatively impair antigen processing, it should be carefully studied to what degree Tn-glycosylation can be introduced to increase uptake via MGL without interfering with antigen presentation.^179^ Rhesus macaques vaccinated with Tn-MUC1 showed significantly higher IFN-γ^+^ T cell responses by ELISpot than those vaccinated with Tn-negative MUC1. Unfortunately, the authors did not investigate whether these were CD4^+^ or CD8^+^ T cells.^180^ In a different study, Tn-MUC6 glycoprotein was found to induce lower IFN-γ but higher IL-17 secretion by CD4^+^ T cells compared to nonglycosylated MUC6. This suggests that targeting of Tn via MGL can promote a Th17 phenotype.^179^

### 4.4 Langerin

Langerin (Clec4k, CD207) is specifically expressed on Langerhans cells (LCs), a skin resident cell population of monocytic origin that displays DC-like properties. LCs patrol dermal tissue, take up antigens and migrate to lymph nodes upon activation.^54,181^ LCs can be targeted by transdermal vaccines through simple application on the skin, which has the advantage to be noninvasive.^182^ Langerin is also expressed at low levels on a subset of murine DC1s and human DC2s. Antigens taken up via Langerin are internalized into Birbeck granules, which are unusual organelles involved in viral degradation.^183^ Langerin has a high affinity for galactose-6-sulfated oligosaccharides and recognizes sulfated and mannosylated glycans.^184^

#### 4.4.1 Langerin–Mouse

In a comparative study, subcutaneous delivery of OVA conjugated to anti-Langerin, anti-DEC205, or anti-DCIR2 revealed that LCs can induce systemic CD4^+^ and CD8^+^ T cell responses.^185^ Anti-Langerin fusions induced fewer CD8^+^ T cells but more CD4^+^ T cells compared to anti-DEC205 fusions upon subcutaneous vaccination, while anti-DCIR2 delivery induced even more pronounced CD4^+^ T cell responses. Moreover, anti-Langerin targeting resulted in a prolonged antigen presentation for several days. Taken together, this indicates that Langerin mediates efficient OVA antigen presentation on both MHC I and MHC II, however this should be confirmed in more clinically relevant settings.^185^ In a different setup, HIV gag-p24 protein was delivered intraperitoneally via anti-Langerin, anti-Clec9a, anti-DCIR2, or anti-DEC205 in combination with anti-CD40 and polyI:C. In comparison with Langerin-targeting vaccines, DC1-targeting vaccines (anti-Clec9a, anti-DEC205) induced comparable IFN-γ-producing CD4^+^ Th1 and CD8^+^ T cells in CB6.F1 mice and outperformed DC2-targeted vaccines (anti-DCIR2).^46^ This suggests that targeting Langerin can be as potent as targeting Clec9a or DEC205. The authors speculate that the evident contradiction between these studies could be due to the model antigen, mouse strain, vaccination route, or adjuvant used. The multitude of possible factors emphasize the potential benefit of standardization of the mouse model (CB6.F1 mice) and vaccine components for comparative therapeutic vaccine studies.

#### 4.4.2 Langerin–Human

Human skin is highly tolerogenic compared with murine skin. Skin resident human LCs are thus more prone to induced peripheral tolerance. Moreover, human LCs have been shown incapable of XP.^186,187^ Langerin can be targeted with glycomimetics of its ligand, which was shown to improve immune responses in various studies when compared to free cargo.^188−190^ However, it remains unclear whether improved pharmacokinetic properties are responsible for this increase or whether Langerin targeting itself played a role. In summary, Langerin might not be an ideal target to induce CD8^+^ T cell-oriented immunity in humans, but rather to direct transdermal vaccines toward LCs in combination with immunostimulatory adjuvants to improve classical antigen presentation to CD4^+^ T cells.

## 5 Promoting Pan-APC Antigen Presentation

### 5.1 DC-SIGN

The main function of DC-SIGN (CD209, Clec4L) is engagement of resting T cells via ICAM-3.^191^ DC-SIGN’s natural ligands are high mannose- and fucose-containing carbohydrates, such as Lewis X oligosaccharides.^192^ DC-SIGN has overlapping ligands with CD206, and displays a similar expression profile.^133,193^ Studying immunity induced by targeting DC-SIGN is complex in mouse models, as mice have eight homologues of human DC-SIGN.^194,195^

#### 5.1.1 DC-SIGN–Human

Targeting antigens to DC-SIGN was shown to induce both CD4^+^ and CD8^+^ immune responses in humanized mice and in vitro models using moDCs.^196−198^ Targeting antigen with anti-DC-SIGN antibodies binding to the carbohydrate recognition domain (CRD) of DC-SIGN promoted routing toward late endosomes, whereas targeting antigens using antibodies binding to the neck region of DC-SIGN routed the antigen to early endosomes.^199^ The latter enhanced T cell proliferation compared to untargeted control; however, the CRD targeting mAb was not investigated for antigen delivery. Still, this study demonstrates that the targeted epitope can influence endosomal processing.

### 5.2 MHC II

MHC II is expressed by all professional APCs. Recycling of MHC II on the cell membrane is rapid in immature DCs but slower in mature DCs leading to a relative higher expression of MHC II on mature DCs.^200^ Targeting antigen to MHC II delivers the antigen to recycling compartments where antigen can be loaded directly on recycled MHC II complexes or cross-presented on MHC I.^201^

#### 5.2.1 MHC II–Mouse

Nanobodies recognizing mouse MHC II conjugated to MUC1 or T cell epitopes of OVA were shown capable of inducing T cell proliferation in B6 mice when administered with immunostimulatory anti-CD40 mAbs and polyI:C.^201,202^ Targeting MHC II increased CD4^+^ T cell activation compared to targeting DEC205. In addition, a MHC II targeted vaccine induced potent humoral immune responses against the SARS-CoV-2 spike protein.^203^ Due to its broad expression on DCs, B cells and other APCs, MHC II might be one of the best ways to reach a large population of immune cells.^204^

### 5.3 CD11c

CD11c (integrin αx, ITGAX) recognizes a variety of ligands, including bacterial lipopolysaccharide (LPS) and several adhesion molecules.^205,206^ It is expressed on all conventional DC subsets as well as several other immune cells such as macrophages, neutrophils, and B cells (Table 1). Conflicting reports have been published on the efficacy of CD11c as vaccine target.^207−211^

#### 5.3.1 CD11c–Mouse

In a comparative study, OVA was targeted to CD11c, DEC205, MHC II, CD40, TLR2, and FcγRII/III by chemical conjugation to the respective Fab′ fragments.^207^ Anti-CD11c conjugates outperformed all others in inducing CD4^+^ and CD8^+^ T cell proliferation in B6 mice. Humoral responses were induced without adjuvant upon targeting CD11c in BALB/c mice.^208^ Furthermore, tumor-reactive CD4^+^ and CD8^+^ T cells were observed in BALB/c mice upon delivery of HER2/neu using anti-CD11c antibody fragments.^209^ In contrast, plasmid DNA vaccination of B6 mice with CD11c-targeted tuberculosis antigen did not increase T cell activation compared to a nontargeting variant, whereas DEC205 targeting did.^210^ It is hypothesized that the vaccine format, as well as the mouse strain, can influence the outcome. We emphasize again the importance of conducting major comparative studies in H2K^b+/d+^ CB6.F1 mice.

### 5.4 CD40

CD40 (Bp50, TNFRSF5) is part of the TNF-receptor superfamily and an important regulator of antigen processing.^212^ Interaction with its ligand CD154 (CD40L) promotes the antigen presentation and maturation of APCs. Agonistic anti-CD40 antibodies have been developed and can be used to mimic the CD40-CD154 interaction. They are utilized as immunostimulatory adjuvant or stand-alone cancer immunotherapy.^213,214^ CD40 is expressed on all APCs and upregulated during maturation.^215^ Similar to DEC205, CD40 is expressed on endothelial cells, which may lead to more off-targeting in comparison with restrictedly expressed DC surface markers.^216^

#### 5.4.1 CD40–Mouse

Agonistic anti-CD40 mAb antigen fusions could be an interesting avenue to combine antigens and adjuvants into a single construct. Fusions of the T cell epitope λ2^315^ of myeloma protein M315 with CD40-targeting single-chain variable fragments (scFvs) induced stronger M315-specific T cell proliferation compared to untargeted fusions.^217^ The agonistic capability of the CD40-targeting moiety was not altered by the conjugation of the antigen in these in vitro experiments. In BALB/c mice, strong IgG2a humoral responses were induced by targeting CD40.^218^ High IgG2a levels are associated with Th1-mediated immunity; thus, targeting antigens to CD40 might have induced a Th1-biased response. Nanoparticles targeted to CD40 induced stronger CD8^+^ T cell responses in comparison with nanoparticles targeted toward DEC205 or CD11c.^219,220^ However, as nanoparticles have pharmacokinetic properties distinct from those of antibody-based vaccines, it would be interesting to repeat this comparative study using antibody-based formulations. Special attention should be given to the intrinsic immunostimulatory capacities of CD40-targeting, as this might provide new opportunities for cancer vaccines without the requirement of systemic adjuvants.

#### 5.4.2 CD40–Human

Enhancement of T cell proliferation and humoral responses by targeting CD40 has been shown for distinct types of antigens (FluM1, HIV, HPV) in vitro.^221−223^ Careful dissection of endosomal routing revealed that targeting CD40 directed antigen to early endosomes, resulting in equally potent XP in comparison with targeting DEC205 on primary DC1s.^47^ Yin et al. performed an extensive comparative study on targeted delivery of MART-1 peptide conjugated to mAb in moDCs.^224^ They observed enhanced MART-1 specific CD4^+^ and CD8^+^ T cell proliferation upon targeting CD40 in comparison to LOX-1 and DECTIN-1. MoDCs were cultured with IL-2 and IL-7 during the T cell activation assay, but no adjuvant was provided. Therefore, it cannot be concluded whether antigen fate or DC maturation induced by CD40 targeting was the main determinant for the enhanced T cell proliferation. Of note, an exciting prospect in the context of CD40 targeting are bispecific antibodies recognizing CD40 and a TAA.^225,226^ These constructs bring tumor debris in proximity to CD40^+^ cells, which can take up, process, and present the tumor antigens, while simultaneously being stimulated via CD40. This results in antitumor responses surpassing immune reactivity toward the initial targeted epitope.^226^

### 5.5 Fc Receptors

Fc receptors (FcRs) are expressed in a variety of immune cells. FcRs recognize specific glycosylation patterns on the Fc-region of mAbs and notably mediate the uptake of opsonized antigens.^227^ They can be actively targeted using anti-CD16, anti-CD32 or anti-CD64 mAbs, or FcR specific ligands. Moreover, FcRs are also passively targeted when mAbs containing functional Fc regions. FcRs are usually specific for a set of isotypes and can trigger inflammatory (e.g., FcγRI, FcγRIIa) or tolerogenic (FcγRIIb) responses.^228^ FcR biology is complex because of their varying affinity for different isotypes and expression patterns on different APCs, and it remains difficult to identify general intracellular pathways. Furthermore, interspecies differences between mouse and human complicate comparison, although signaling cascades and effector function are reasonably conserved.^229^ Unlike most endocytic receptors mentioned in this review, FcRs generally do not release their ligand upon internalization, which has been suggested to target antigens toward lysosomes.^230^

#### 5.5.1 Fc Receptors–Mouse

Formyl peptide receptor-like 1 inhibitor (FLIPr) is secreted by bacteria to evade opsonization by binding antagonistically to FcRs.^231^ Fusions of FLIPr with OVA conferred a significant survival benefit in tumor-bearing B6 mice, even in the absence of an adjuvant.^232,233^ These effects were not observed in TAP^–/–^ B6 mice highlighting the necessity for XP to obtain antitumor effects.^233^

#### 5.5.2 Fc Receptors–Human

Active targeting of FcγRII on moDCs with a peptide derived from human cytomegalovirus enhanced XP compared to nontargeting variants in vitro, demonstrating the possibilities of actively targeting FcRs.^234^ In contrast, antigen delivery via FcεRI-bound IgE targets antigens to a cathepsin S-dependent MHC class II pathway is expected to favor CD4^+^ T cell responses.^235^ The diverse physiological functions of FcRs are reflected in the various types of vaccines targeting them.^236−238^ Examples include vaccines targeting FcγRI for protection against dengue, vaccines recruiting intracellular FcR TRIM21 to induce XP in moDCs, and vaccines targeting multiple FcRs (CD16, CD32, CD64) to dampen autoimmunity. FcRs can be targeted to promote classical antigen presentation or XP, but the specific pathways activated by targeting different FcRs require further investigation for the therapeutic vaccine development.

## 6 Future Perspectives and Recommendations for Therapeutic Vaccine Design

### 6.1 Controlling the Immune Response by Targeting Specific DC Receptors

Therapeutic vaccines have yet to achieve the same impact on modern healthcare as prophylactic vaccines. While it is firmly established that targeting vaccines to DC receptors enhances therapeutic efficacy, the optimal target remains elusive. Here, we reviewed the literature regarding distinct receptors for their effect on antigen presentation and immune response (Table 2).

Targeting therapeutic vaccines to DC1-specific receptors harnesses the superior ability of DC1s at XP and improves CD8^+^ T cell responses.^50^ The first-in-human DC-targeted vaccine CDX-1401 demonstrated a reasonable safety profile and a proof-of-concept for feasibility. It elicited NY-ESO-1-specific T cell responses in patients, but yielded only modest clinical benefits.^99,101^ The observed T cell responses are likely not a result of increased XP directly but rather increased uptake by DCs in general. The broad expression pattern of DEC205 on myeloid and nonmyeloid cells may have hindered efficient delivery of NY-ESO-1 to DC1s. Targeting of the DC1-specific receptors Clec9a or XCR1 induced stronger XP in preclinical studies in comparison with human DEC205, which we hypothesize is due to fundamental differences in intracellular routing.^44,106,111,112^ We envision that targeting Clec9a or XCR1, both exclusively expressed on cross-presenting DC1s, could strongly improve the efficacy of therapeutic cancer vaccines through the promotion of potent XP of the delivered antigen. Importantly, CDX-1401 efficacy was potentiated by pretreatment with Flt3l, which increased the abundance of peripheral conventional DCs and monocytes.^101^ Other studies including Flt3l have shown its ability to induce differentiation and recruitment of DC1s.^239^ Therefore, pretreatment with Flt3l could be especially beneficial to vaccines designed to improve XP by targeting of DC1s. Yet, the low numbers of circulating DC1s (<0.05% of PBMCs) could restrict the absolute antigen uptake, and therefore targeting a larger fraction of APCs may overall be more beneficial. Antigen retention and transfer to DC1s by CD169^+^ macrophages is a promising strategy to improve antigen XP.^126,128^ Finally, targeting CD206 was shown to promote XP by delivering antigens to early endosomes.^135,136^ Because CD206 is also expressed on TAMs, antigens should only be targeted to CD206 in combination with TAM repolarization agents such as TLR7/8-agonists.^140^ Clinical studies exploring antigen targeting to CD206 (e.g., CDX-1307) may especially benefit from codelivery of adjuvants.^138,139^

Antigen routing toward late endosomes or direct delivery in the MHC II loading compartment improves CD4^+^ Th responses and humoral immunity. This can be promoted by targeting DC receptors such as DCIR(2), MGL(2), or MHC II. Incorporating CD4^+^ T cell epitopes in vaccine design is expected to support cytotoxic immune responses by means of CD4^+^ T cell help.^40,62^ Finally, the immunostimulatory signaling inherent by receptors as DECTIN-1 or CD40 could be advantageous when trying to break tolerance to self-antigens and should be further explored in the context of therapeutic cancer vaccines.^138,156,158^

### 6.2 Specific Targeting of DC Subpopulations or Broad Targeting of pAPCs?

Targeting FcRs or MHC II allows for the targeting of a broad range of APCs, and this approach has been shown to significantly enhance immune responses.^202,203,234^ Targeting a larger pool of APCs could potentially elicit an immune response of greater magnitude compared to targeting less abundant DC subpopulations.^24^ Yet, broad targeting approaches could be more prone to induce Tregs if antigens are delivered to immune suppressive APCs such as TAMs. So far, there have been no reports of a direct comparison between therapeutic cancer vaccines that target broadly expressed receptors, such as MHC II or FcR and vaccines that target receptors with restricted expression, such as XCR1 or Clec9a. Such comparative studies would help define whether strict targeting or broader targeting induces optimal antitumor immune responses to explore for clinical translation. Lastly, combined targeting of CD8 epitopes to XP-enhancing receptors and CD4 epitopes to receptors specialized in class II antigen presentation may be of interest to pursue in the field of therapeutic cancer vaccines.

### 6.3 Extensive Characterization of Adjuvants, Immune Responses, and Formulation

Immunostimulatory adjuvants are essential in therapeutic cancer vaccines to induce immune responses toward the presented epitope. Antigen presentation, in absence of immunostimulatory adjuvants, by immature DCs induces tolerance and tumor progression.^240,241^ Preferably, antigen and adjuvant should be delivered simultaneously to DCs, as this increased antigen presentation in multiple models.^87,88^ A premature maturation of DCs could impair vaccine uptake and cause autoimmunity in response to systemic adjuvant encounter.^4^ Covalent incorporation of adjuvants into DC-targeted vaccines could improve the therapeutic window, ensure simultaneous delivery of both antigen and adjuvant and limit off-target effects.^27,29,242,243^ Much like interspecies differences in DC surface receptors, variations in TLR expression patterns affect the clinical translation of murine studies.^3,244^ For instance, murine DC1s do not express TLR7, yet human DC1s are highly responsive to imidazoquinolines, a class of TLR7-agonists with promising therapeutic potential.^245^ TLR3 is mainly expressed by DC1s, which would indicate that TLR3-agonists as poly-I:C can be more effective than other adjuvants to promote XP and CD8^+^ T cell activation by DC1s. A combination therapy of TLR3-agonist, Flt3l, and radiotherapy recruited cross-presenting DCs in the tumor, resulting in cytotoxic T cell responses and tumor clearance in lymphoma patients.^239^ These promising results emphasize the potential of Flt3l to stimulate DCs, which could also be used in combination with therapeutic vaccines.

The significant role of CD4^+^ T cells in driving antitumor responses is increasingly evident.^40,41,62,246^ While most mechanistic studies focus on individual pathways, we recommend including the characterization of T helper responses and humoral responses along with cytotoxic T cell responses. Such studies will be crucial to delineate whether a single receptor on DCs can optimally induce XP as well as classical presentation or whether combination therapies should be investigated as an ideal targeting strategy. In mouse studies, the strain should be considered carefully.^164,165^ We recommend the use of CB6.F1 mice to unify previous findings, as this hybrid mouse strain neutralizes the difference between the commonly used B6 and BALB/c mice. While moDCs are easy to generate at moderate costs and well-suited for initial studies, they have inherent differences in antigen processing and presentation capacity, and are therefore not fully reflective of circulating primary DCs.^21,22^ Therefore, in vitro studies should include naturally occurring DC subsets to confirm findings on moDCs. Finally, the influence of the isotype in the case of a targeting mAb should be studied to account for FcR-mediated effects.^227,228,247^ We emphasize the importance of including isotype controls in DC targeted vaccine studies. Improved circulation time has been shown to positively affect targeting and improve immunological outcome of targeted vaccines.^110^ Fc-isotype engineering could offer opportunities for improving circulation time, for example through incorporation of Fc-silent mutations or increasing affinity to neonatal FcR to promote recycling of mAbs.^248−250^

This review focuses on targeted antigen delivery through antibody and ligand conjugates. Targeting of DCs has also been explored using other delivery vehicles, for instance, nanoparticles or viral vectors. Nanoparticle encapsulation of antigens is an attractive strategy to extend half-life and allow codelivery of antigens and adjuvants. The particulate nature renders them especially susceptible to phagocytosis by APCs. However, this nature also promotes clearance via neutrophils or macrophages, reducing antigen presentation. Although DC targeting of nanoparticles can be improved by conjugation of antibodies or ligands recognized by DC receptors, skewing the biodistribution of nanoparticles is difficult, as it is mostly dictated by particle size and surface composition.^251^ Larger nanoparticles (>150 nm in diameter) are cleared by the reticuloendothelial system and taken up by phagocytic cells in proximity of the injection site or in the liver. In contrast, smaller nanoparticles (<150 nm in diameter) can evade clearance by the reticuloendothelial system and reach lymph nodes.^252^ A positive surface charge has been reported to improve the uptake of nanoparticles in vivo.^253,254^ For more in-depth reading on nanoparticles vaccination strategies, we refer to reviews covering this subject.^255−258^

An approach different from nanoparticles or antibody or ligand conjugates is viral vectors. Lentiviral vectors are a commonly used type of vector to deliver antigens to DCs, which are inherently immunogenic, thereby also serving as adjuvant. Engineering of the Sindbis viral vector by removing the heparan sulfate recognition site, while preserving the glycoprotein recognized by DC-SIGN, enables targeting of these vectors toward DCs.^259^ Phase I/II clinical trials for the DC-SIGN targeted lentiviral vector LV305 containing a NY-ESO-1 TAA demonstrated the feasibility of this approach, as no undesired mutagenesis and viral persistence were noted.^260,261^ Direct comparisons between delivery vehicles are seldom performed. This is an interesting avenue to explore and could provide new insights into the field of targeted antigen delivery.

### 6.4 Recommendations for Designing Targeted Therapeutic Cancer Vaccines

In this Review, we suggest that selecting the appropriate target may surpass selecting a specific DC subset. Distinct receptors on a single DC subset engage specific endosomal pathways, resulting in different levels of antigen presentation. An ideal therapeutic cancer vaccine achieves sufficient XP to prime CD8^+^ T cells, while maintaining adequate CD4^+^ T helper activation. The next step in targeted therapeutic vaccination is the translation of preclinical studies into the clinic. We recommend the use of Clec9a or XCR1 as target for therapeutic vaccines in combination with Flt3l to boost DC1 abundance.^101^ Targeting Clec9a or XCR1 results in superior CD8^+^ T cell mediated antitumor immunity over targeting DEC205, while sustaining CD4^+^ T helper cell responses in preclinical models.^44,46,110−112^ Moreover, Clec9a and XCR1 are specifically expressed on DC1s, thereby limiting off-target engagement. Flt3l coadministration could augment DC1-targeted therapies by increasing the natural low abundance of DC1s (<0.05% of PBMCs), as demonstrated by clinical trials administering CDX-1401 in combination with Flt3l.^101^ To further enhance vaccine efficacy, we would recommend incorporation of immunostimulatory adjuvants, for example through encapsulation in nanoparticles, antigen-adjuvant conjugates, or self-adjuvating mRNA vaccines.^29,242^ This will ensure simultaneous delivery of adjuvant and antigen to DCs, which strongly improves antigen presentation, reduces tolerance induction, and may improve the therapeutic window of the adjuvant by reducing systemic immune activation.^87,88^

Results of clinical trials have shown the safety and feasibility of in vivo cancer vaccination (Table 2). Thorough preclinical evaluation and comparison of different vaccines and targets is required to move toward clinical translation. It will require head-to-head comparisons of different vaccine targets and experimental standardization, for example, in terms of the use of mouse strain, adjuvant, and isotype controls. Studies on human targets should be performed on primary DCs as much as possible to aid the clinical translation of the vaccine formulation. It is recommended that future studies focus on a broad readout of the immune response including phenotypic characterization of CD4^+^ and CD8^+^ T cell responses.^62,246^ An interesting comparative study would be to investigate side-by-side targeting of broadly expressed receptors, such as MHC II or FcRs, and restrictedly expressed receptors, such as Clec9a or XCR1. This will help to establish the most optimal target to pursue in therapeutic cancer vaccines. It is well established that targeting MHC I epitopes toward receptors promoting XP enhances CD8 T cell responses in a preclinical setting. However, analyzing this in a clinical setting remains challenging. Humanized mouse models offer an opportunity to assess the rationale of steering the immune system through receptor targeting, yet capturing the full complexity of the human immune system will be difficult. Therefore, the true benefits of novel targeted antigen delivery strategies ought to be assessed in clinical trials, which is an exciting prospect. To conclude, ample suitable surface markers on DCs exist for targeted therapeutic cancer vaccines, and proper selection will undoubtedly enhance future vaccine efficacy.

## Abbreviations

APC: antigen-presenting cell
DC: dendritic cell
MHC: major histocompatibility complex
PRR: pathogen recognition receptor
TLR: Toll-like receptors
DAMP: damage associated molecular pattern
PAMP: pathogen associated molecular pattern
iTregs: induced regulatory T cells
TAA: tumor-associated antigen
gp100: glycoprotein 100
TRP-2: tyrosine related protein 2
TSA: tumor-specific antigen
NK: natural killer
moDC: monocyte-derived dendritic cell
DC1: type 1 conventional dendritic cell
DC2: type 2 conventional dendritic cell
HPV: human papillomavirus
XP: cross-presentation
ER: endoplasmic reticulum
ETP: endosome transporter protein
UPS: ubiquitin-proteasome system
TAP: transporter associated with antigen processing
ERC: endosomal recycling compartment
pDC: plasmacytoid dendritic cell
Th1: T helper type 1
Th2: T helper type 2
cDC: conventional dendritic cell
OVA: ovalbumin
mAb: monoclonal antibody
Flt3L: fms-like tyrosine kinase 3 ligand
HA: hemagglutinin
BMDC: bone marrow-derived dendritic cell
hCG-β: Human chorionic gonadotropin β
TAM: tumor-associated macrophage
KLH: keyhole limpet hemocyanin
GalNAc: galactosamine
Tn: Thomsen nouveau
LC: Langerhans cell
CRD: carbohydrate recognition domain
LPS: lipopolysaccharide
scFv: single chain variable fragment
FLIPr: formyl peptide receptor-like 1 inhibitor
